# Economic Disadvantage and Young Children’s Emotional and Behavioral Problems: Mechanisms of Risk

**DOI:** 10.1007/s10802-012-9655-2

**Published:** 2012-06-27

**Authors:** Jolien Rijlaarsdam, Gonneke W. J. M. Stevens, Jan van der Ende, Albert Hofman, Vincent W. V. Jaddoe, Johan P. Mackenbach, Frank C. Verhulst, Henning Tiemeier

**Affiliations:** 1 Department of Child and Adolescent Psychiatry, Erasmus MC-University Medical Center Rotterdam, Rotterdam, the Netherlands; 2 The Generation R Study Group, Erasmus MC-University Medical Center Rotterdam, Rotterdam, the Netherlands; 3 Interdisciplinary Social Sciences, Faculty of Social Sciences, University of Utrecht, Utrecht, the Netherlands; 4 Department of Epidemiology, Erasmus MC-University Medical Center Rotterdam, Rotterdam, the Netherlands; 5 Department of Paediatrics, Erasmus MC-University Medical Center Rotterdam, Rotterdam, the Netherlands; 6 Department of Public Health, Erasmus MC-University Medical Center Rotterdam, Rotterdam, the Netherlands; 7 Department of Psychiatry, Erasmus MC-University Medical Center Rotterdam, Rotterdam, the Netherlands

**Keywords:** Poverty, Child behavior problems, Maternal depression, Parenting, Home environment, Prospective study

## Abstract

This study aimed to establish potential mechanisms through which economic disadvantage contributes to the development of young children’s internalizing and externalizing problems. Prospective data from fetal life to age 3 years were collected in a total of 2,169 families participating in the Generation R Study. The observed physical home environment, the provision of learning materials in the home, maternal depressive symptoms, parenting stress, and harsh disciplining practices were all analyzed as potential mediators of the association between economic disadvantage and children’s internalizing and externalizing problem scores. Findings from structural equation modeling showed that for both internalizing and externalizing problems, the mechanisms underlying the effect of economic disadvantage included maternal depressive symptoms, along with parenting stress and harsh disciplining. For internalizing but not for externalizing problem scores, the lack of provision of learning materials in the home was an additional mechanism explaining the effect of economic disadvantage. The current results suggest that interventions that focus solely on raising income levels may not adequately address problems in the family processes that emerge as a result of economic disadvantage. Policies to improve the mental health of mothers with young children but also their home environments are needed to change the economic gradient in child behavior.

## Introduction

It has been widely acknowledged that poverty has a harmful impact on children’s development (Bradley and Corwyn 2002; Brooks-Gunn and Duncan 1997; McLoyd 1998). Children residing in economically deprived families more often manifest behavioral and emotional problems (Bradley and Corwyn 2002). In addition, there is evidence that the harmful effects of poverty are already observable early in a child’s life. For example, studies in the United States and the United Kingdom have shown that associations of low income with children’s behavior and emotional well-being occur when children are as young as age 3 and 5 years (Kiernan and Huerta 2008; Linver et al. 2002; Yeung et al. 2002). Given these findings, interventions during the early years of a child’s life may be most important in diminishing the harmful effects of poverty on children’s behavioral and emotional development (Brooks-Gunn and Duncan 1997).

However, without information on the mechanisms underlying the association between poverty and adverse child development, leverage points amenable to policy intervention are unclear, leaving professionals and policy makers with little information on how to guide these interventions. There is ample evidence that the home environment and parental emotional well-being mediate the association between low family income and child emotional and behavioral problems (Bor et al. 1997; Kiernan and Huerta 2008; Linver et al. 2002; McLeod and Shanahan 1993; NICHD 2005; Pachter et al. 2006; Yeung et al. 2002). Using data from the National Longitudinal Survey of Youth, Pachter et al. (2006) found that poverty affected children’s behavioral and emotional problems from the age of 6 to 9 years through more proximal variables such as maternal depression and the child’s home environment. Yeung et al. (2002) found that children residing in families with lower income had more behavioral problems, and this effect was partially mediated by the quality of the child’s home environment, maternal depressive symptoms, and parenting quality. Their results demonstrated that family income was directly associated with maternal depressive symptoms, but also indirectly, through the physical home environment. Maternal depressive symptoms were in turn associated with punitive parenting, which was then related to children’s behavioral problems (Yeung et al. 2002).

The results of Yeung et al. (2002) are consistent with the family investment model and the family stress model that have been proposed to explain mechanisms connecting socioeconomic status (SES) and behavioral development (Conger and Elder 1994). Conger and his colleagues have postulated that economic disadvantage is negatively related to parental material investments in the development of children (RD Conger and KJ Conger 2002; Conger and Donnellan 2007; Conger and Elder 1994; Martin et al. 2010). These investments in children involve dimensions of family support, such as stimulation of learning and adequate housing. In addition, Conger and his colleagues have proposed that economic disadvantage adversely affects the child’s development through its negative impact on parental emotional well-being such as depression, which in turn diminishes or disrupts parenting skills (RD Conger and KJ Conger 2002; Conger and Donnellan 2007; Conger and Elder 1994; Martin et al. 2010). In line with expectations derived from the family investment model, research has shown that children residing in poor families have limited access to age-appropriate learning resources (e.g., learning toys or books) in the home, and are more likely to live in houses with structural defects (Bradley and Corwyn 2002; Duncan and Brooks-Gunn 2000; Evans 2004; McLoyd 1998). Consistent with predictions from the family stress model, economic disadvantage has been related to maternal depression, which predicts disruptions in parenting including more harsh disciplinary practices and parenting stress (Forman et al. 2007; Goodman and Brumley 1990; Lovejoy et al. 2000; McLoyd 1998).

The vast majority of studies investigating mechanisms underlying the association between low income and young children’s emotional and behavioral problems have been conducted in the United States. In the United States, economic inequalities are more pronounced than in any other industrialized nation (Caminada and Goudswaard 2001; Moss 2000) and economic mobility for those in poverty is among the lowest (Belle and Doucet 2003). Whereas the essence of US antipoverty policies is to indirectly approach poverty reduction by providing poor families with education and support services, European interventions seek to provide social insurance programs (e.g., universal health care) and programs that directly raise incomes of poor families (e.g., minimum wage) (McLoyd 1998; Moss 2000). Associations between low income and children’s development exist in such publicly funded health-care systems but these associations tend to be weaker (Propper et al. 2007).

There may also be differences between the United States and other wealthy nations in the specific mechanisms by which low income influences children’s well-being. Income inequality has been strongly related to depression, particularly among women with young children (Belle and Doucet 2003; Kahn et al. 2000). High levels of depressive symptoms are common in the United States; recent estimates suggest that the 12-months prevalence of major depressive disorder in mothers is 10.2 % (Ertel et al. 2011). Thus, in a family stress based model explaining the effects low income has on the child’s development, associations involving maternal depressive symptoms may be absent or less strong in nations with less income disparities. Despite these potential differences, few studies extended this research on economic disadvantage and young children’s emotional and behavioral development to nations other than the United States.

A notable exception is the recent study of Kiernan et al. (Kiernan and Huerta 2008) that used data from the UK Millennium Cohort Study to examine associations between economic deprivation and child emotional and behavioral problems at the age of 3 years. The authors reported that economic deprivation was related to children’s emotional and behavioral problems and that these associations were partially explained by maternal depressive symptoms and parenting factors such as disciplining practices. However, such efforts remain rare and additional research that addresses family processes underlying the impact of economic disadvantage on a non-USA sample of children is needed.

**Fig. 1:**
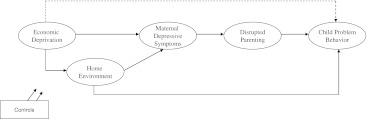
Conceptual model. The conceptual framework of our proposed model in which the quality of children’s home environments, maternal depressive symptoms, and disrupted parenting mediate the association between economic disadvantage and child problem behavior

The current study assessed whether the mediational processes by which economic disadvantage is proposed to affect child emotional well-being and behavioral problems held true for a Dutch sample of young children. Using data from a population-based prospective study, we examined children’s home environments, maternal depressive symptoms, and disruptions in parenting as potential mediators of the association between economic disadvantage and children’s emotional and behavioral problems. We investigated two different dimensions of disrupted parenting: mother’s harsh disciplining and parent related parenting stress (i.e., mother’s attitudes toward her parenting). In addition, we focused on two dimensions of the home environment: the physical home (e.g., housing quality) and the provision of learning materials and toys in the home. This allowed us to examine predictions from both the family stress model and the family investment model. We hypothesized that family investments (as indicated by home environments) and family stress (as indicated by maternal depressive symptoms or disrupted parenting) constitute non-exclusive mechanisms that explain the association between economic disadvantage and children’s behavioral development. Firstly, we hypothesized that economic disadvantage directly affects the quality of home environments and maternal depressive symptoms. Maternal depressive symptoms in turn disrupt parenting, which then has an effect on young children’s behavioral development. Secondly, we hypothesized that home environments are also directly related to children’s behavioral development. Finally, we postulated that home environments are indirectly related to children’s behavioral development through maternal depressive symptoms and disrupted parenting. Figure 1 represents the conceptual framework of our proposed model.

## Method

### Study Design

The present study was conducted within Generation R, a longitudinal, population-based cohort from fetal life onwards (Jaddoe et al. 2010). Pregnant women living in the study area in Rotterdam, the Netherlands, with an expected delivery date between April 2002 and January 2006, were invited to participate. Written informed consent was obtained from all participants. The study was conducted in accordance with the guidelines proposed in the World Medical Association Declaration of Helsinki, and was approved by the Medical Ethics Committee of the Erasmus Medical Center, Rotterdam (numbers: prenatal, MEC 198.782/2001/31 and postnatal, MEC 217.595/2002/202).

### Population for Analysis

This study was embedded in the postnatal phase of the Generation R Study, which was constituted with a renewed consent procedure when infants were around 3 months of age. In 3,400 children this consent procedure was combined with a home visit which included an observation of the home environment. We excluded twins, leaving 3,334 children eligible for follow-up. A total of 2,169 mothers reported on child behavior at the 3-year assessment. These 2,169 children and their families (65 % of 3,334) were included in the current analyses. In order to test consistency, we also used father reports of child behavior. A total of 1,621 fathers in this sample reported on child behavior at the 3-year assessment.

In the current sample of 2,169 children and their families, the mean age of children at the behavioral assessment was 36.58 months (*SD* = 1.22). Forty eight percent of the children in this sample were boys. Thirty one percent of children were non-Western, and 47 % were first born. The mean age of mothers in this sample at intake was 31.38 years (*SD* = 4.66). General secondary school was the highest educational level attained in 44 % of the mothers. Of the mothers, 9 % were single.

Respondents (*n* = 2,169) were more often of Western national origin (69.2 % vs. 44.4 %, *χ* ^2^ = 175.03, *p* < 0.001) and were less often poor (12.9 % vs. 37.7 %, *χ* ^2^ = 198.22, *p* < 0.001) than non-respondents (*n* = 1,165). Respondents more often completed higher levels of education than non-respondents (56.5 % vs. 26.2 %, *χ* ^2^ = 240.11, *p* < 0.001).

### Measures

Our analysis included child internalizing and externalizing problem behavior assessed at the age of 3 years, economic disadvantage assessed at 30 weeks of gestation, and five mediators (the physical and the stimulating home environment assessed at the age of 3 months, maternal depressive symptoms assessed at the age of 6 months, parenting stress assessed at the age of 1.5 years, and harsh disciplining assessed at the age of 3 years). We examined all these variables via latent constructs. Table 1 presents the variables that were used as indicators of the latent constructs. The first column of Table 1 displays the means or percentages of these variables.

**Table 1:** Summary of confirmatory factor analysis measurement models

Measurement model	% yes/Mean (*SD*)^a^	Estimates^b^	
Model 1: Children’s outcomes			
Internalizing			
Emotionally reactive	1.62 (1.83)	1.00^c^	(0.00) **0.83**
Anxious or depressed	1.09 (1.54)	0.74***	(0.03) **0.63**
Somatic complaints	1.63 (1.75)	0.62***	(0.03) **0.53**
Withdrawn behavior	0.95 (1.37)	0.64***	(0.03) **0.59**
Externalizing			
Attention	1.48 (1.62)	1.00^c^	(0.00) **0.64**
Aggressive	7.00 (5.16)	1.96***	(0.07) **0.90**
Internalizing with Externalizing		0.28 ***	(0.01) **0.83**
CFI = 0.97; TLI = 0.95; RMSEA = 0.08; *χ* ^2^(8) =123.18			
Model 2: Economic disadvantage			
Low income	13	1.00^c^	(0.00) **0.69**
Financial difficulties	18	1.22***	(0.06) **0.84**
Not having friends or family over for dinner	2	1.29***	(0.07) **0.89**
No evening out once every two weeks	8	1.29***	(0.07) **0.89**
No holiday from home	8	1.33***	(0.07) **0.92**
No membership of a social or cultural club	5	1.34***	(0.07) **0.92**
No leisure items	3	1.27***	(0.07) **0.87**
No regular purchase of new clothes	10	1.23***	(0.07) **0.84**
Postponed payment of rent or mortgage	2	1.05***	(0.08) **0.72**
No car or lease car	5	1.12***	(0.07) **0.77**
CFI = 0.99; TLI = 0.99; RMSEA = 0.03; *χ* ^2^(25) = 68.60			
Model 3: Home environment			
Physical home			
Street is clean	89	1.00^c^	(0.00) **0.83**
Exterior of the house is well maintained	93	1.10***	(0.04) **0.91**
Neglected houses in the street^d^	87	0.99***	(0.04) **0.81**
Basic furniture is present	97	0.79***	(0.06) **0.65**
Windows or walls are damp inside^d^	96	0.85***	(0.06) **0.70**
The walls inside the house are in good condition	94	0.96***	(0.05) **0.79**
Central heating system is present	95	0.61***	(0.06) **0.51**
The living room is tidy	79	0.70***	(0.04) **0.58**
The kitchen or toilet is unclean^d^	79	0.78***	(0.04) **0.65**
Cigarette smoke in the residence^d^	96	0.60***	(0.07) **0.50**
Stimulating home			
Various toys	87	1.00^c^	(0.00) **0.99**
Special place to lay down and play	90	0.89***	(0.02) **0.88**
Cuddly toys are available	86	0.97***	(0.01) **0.96**
Muscle activity toys or equipment	83	0.94***	(0.01) **0.94**
Musical toys or equipment	84	0.94***	(0.01) **0.94**
Physical home with Stimulating home		0.34***	(0.03) **0.41**
CFI = 0.97; TLI = 0.97; RMSEA = 0.07; *χ* ^2^(40) = 495.21			
Model 4: Maternal depressive symptoms			
Feeling suicidal	3	1.00^c^	(0.00) **0.83**
Feeling lonely	26	0.95***	(0.06) **0.79**
Feeling down	23	1.05***	(0.06) **0.87**
Having no interest	13	1.00***	(0.06) **0.83**
Feeling desperate about the future	17	1.04***	(0.06) **0.87**
Feeling worthless	11	0.98***	(0.06) **0.81**
CFI = 1.00; TLI = 1.00; RMSEA = 0.02; *χ* ^2^(8) = 12.60			
Model 5: Parenting			
Parenting stress			
Being a parent is difficult	27	1.00^c^	(0.00) **0.68**
Trouble raising child	19	1.23***	(0.05) **0.83**
Thinking about giving up	9	1.14***	(0.06) **0.77**
Not capable of caring for child	5	1.24***	(0.06) **0.84**
Difficulties making decisions about child	8	0.96***	(0.06) **0.65**
Not being able to cope with things	16	1.19***	(0.05) **0.80**
Getting tired quickly	78	0.49***	(0.06) **0.33**
Feeling not to have things under control	23	1.15***	(0.05) **0.77**
Wanting to be a mother like that	16	0.97***	(0.05) **0.65**
I often do not understand my child	15	0.90***	(0.06) **0.60**
I am not confident about the future upbringing	26	0.61***	(0.06) **0.41**
Harsh disciplining			
I shook my child	7	1.00^c^	(0.00) **0.74**
I shouted or screamed angrily at my child	76	0.92***	(0.08) **0.68**
I called my child names	5	1.14***	(0.09) **0.84**
I threatened to give a slap but I didn’t do it	30	0.67***	(0.06) **0.50**
I angrily pinched my child’s arm	15	0.74***	(0.07) **0.55**
I called my child stupid or lazy or something like that	7	1.01***	(0.08) **0.75**
Parenting stress with harsh disciplining		0.21***	(0.03) **0.43**
CFI = .97; TLI = .98; RMSEA = 0.03; *χ* ^2^(83) = 218.40			

*CFI* Comparative fit index; *TLI* Tucker-Lewis index; *RMSEA* Root mean square error of approximation

^a^Values represent mean (*SD*) for continuous indicator variables and percentages for categorical indicator variables

^b^Unstandardized and standardized (bold) coefficient estimates (values given in parentheses are standard errors)

^c^According to requirements for structural equation modeling one variable loading on each latent factor was set equal to 1.00 to set the metric for that factor. Consequently, significance values are not calculated for these variable loadings

^d^Reversed items were recoded prior to analysis

*** *p* < 0.001

#### Child Behavior

The Child Behavior Checklist for toddlers (CBCL/1,5-5; Achenbach and Rescorla 2000) was used to obtain standardized reports of children’s problem behavior at the age of 3 years. The CBCL includes 99 items on which parents rate the extent to which each statement describes their child “now or within the past 2 months” on a three point scale; 0=*not true*, 1=*somewhat or sometimes true* and 2=*very true or often true*. In the present study, six CBCL syndrome scales were used, to index emotionally reactive behavior (9 items), anxious or depressed behavior (8 items), somatic complaints (11 items), withdrawn behavior (8 items), attention problems (5 items) and aggressive behavior (19 items). Scale scores were computed by summing respective items. The psychometric properties of the CBCL are well established (Achenbach and Rescorla 2000). The CBCL syndrome scales were square root transformed for the current study to approximate a normal distribution.

#### Economic Disadvantage

The economic disadvantage construct included several measures collected at 30 weeks of gestation: family income, financial difficulties, and adjustments the family had to make because of financial difficulties (see Table 1). Primary caretakers were asked to report the total monthly net *income* of their household. To compare poor and non-poor families, we dichotomized our measure of family income in accordance with the social security level (above (≥ €1200) versus below the social security level (< €1200)). Individuals living below social security level are considered to have insufficient means to acquire immediate needs and are entitled to apply for social security benefits. Primary caregivers also reported whether they experienced *financial difficulty* in acquiring immediate needs such as food, rent and electricity in the last year. Responses were coded as 0=not difficult; 1=difficult. In addition, primary caregivers reported on 13 *adjustments* the family had to make in the last year because of financial difficulties. For example, caregivers were asked whether they regularly purchased new clothes. For negative answers, a follow-up question examined whether this adjustment was made because of financial difficulties. Due to very low prevalence rates (≤0.5 %) and estimation problems that were encountered because of empty cells, five of these follow-up items were removed from analyses (i.e., at least one warm meal a day, adequate heating, having a refrigerator, a telephone, or a washing machine at home). In order to address upward mobility (i.e., families may increase wealth several years later), we also included a family income measure collected when the child was 3 years of age.

#### Home Environments

Home environments were assessed by means of observation during a home visit when the infant was on average 3.37 months of age (*SD* = 1.15). The physical home environment construct was derived from ten binary-coded items from the adapted IT-HOME Inventory (Rijlaarsdam et al. 2012), registering, among other things, whether the home was clean or whether a central heating system was present (see Table 1). The stimulating home environment construct was derived from five binary-coded items from the adapted IT-HOME Inventory assessing, among other things, whether the infant had musical toys (see Table 1). These five items were guided by the Infant-Toddler Home Observation for Measurement of the Environment Inventory (IT-HOME; Caldwell and Bradley 1984). Good reliability has been demonstrated for the adapted IT-HOME Inventory (Rijlaarsdam et al. 2012).

#### Depressive Symptoms

The construct of maternal depressive symptoms was derived from the six items of the depression scale from the Brief Symptom Inventory (BSI; De Beurs 2004; Derogatis 1993) collected when the child was 6 months of age. The BSI is a validated self-report measure consisting of 53 items, which is widely used in clinical and research settings. The items define a spectrum of depressive symptoms such as “feeling lonely” in the preceding 7 days and are rated on 5-point uni-dimensional scales, ranging from 0 (*not at all*) to 4 (*extremely*).

#### Parenting Stress

The parenting stress construct included 11 items of the parenting domain of the Nijmeegse Ouderlijke Stress Index-Kort (NOSIK; De Brock et al. 1992) collected when the child was 1.5 years of age. The NOSIK is the Dutch version of the Parenting Stress Index-Short Form (Abidin 1983). Sample parenting stress items include “I often do not understand my child” and “Being a parent is difficult”. Items were rated on a 4-point Likert scale. Higher scores indicate greater levels of stress. Good reliability (Cronbach’s alpha = 0.95) and validity have been reported for the NOSIK (De Brock et al. 1992).

#### Disciplining Practices

The harsh disciplining construct was derived from six items of the Parent–child Conflict Tactics Scale (CTS; Straus et al. 1998) collected when the child was 3 years of age. Mothers rated their disciplining practices during the past 2 weeks on a 6-point scale ranging from 0 (*never*) to 5 (*five times or more*). Examples of questions are “I shouted or screamed angrily at my child” and “I angrily pinched my child’s arm” (see Table 1).

#### Family Sociodemographics

Information on family socio-demographic characteristics was obtained by questionnaire during pregnancy. We included as covariates in our analyses child gender, child’s age at the assessment of outcome, parity (previous pregnancies: 0 versus ≥1), maternal age at intake, marital status (married or cohabiting versus single), and mothers’ highest attained educational level (no formal education completed or general secondary education versus higher vocational training or higher academic education), and child national origin. Child national origin was classified into Western versus non-Western and was based on the country of birth of the parents. The group classified as Western includes European, North-American, Australian, and Asian Western (Japanese) children. The non-Western group is comprised of children with a Turkish, Moroccan, Surinamese, Cape Verdian, Dutch Antillean, African, South-American, and Asian non-Western (Asia except Japan) national origin.

### Statistical Analysis

The analyses were conducted in Mplus version 5.1 (Muthén and Muthén 1998–2007). Missing data were estimated in order to use all available data in Mplus with full information maximum likelihood (FIML) procedures as described by Asparouhov and Muthén (2010). First, the factor structures of the predictor, outcome, and mediator variables were tested to confirm that these measures show good psychometric properties in the current sample. This was accomplished by conducting five confirmatory factor analyses (CFA); (1) children’s outcomes including internalizing and externalizing problems, (2) economic disadvantage, (3) home environments including the physical and the learning home environment, (4) maternal depressive symptoms, and (5) parenting including parenting stress and harsh disciplining.

We determined identification of all CFA measurement models. For example, the internalizing and externalizing model was identified by the two-indicator rule (e.g., Kline 2011): (a) there is more than one factor, (b) there are two or more indicators per factor, (c) the two factors are allowed to covary, and (d) theta is diagonal, which means that there are no correlated errors in indicators. The Maximum Likelihood estimator was used for the internalizing and externalizing CFA measurement model, which is the default in Mplus for analysis with all continuous variables. Categorical items were recoded to be dichotomous (0=never or not true and 1=yes, any endorsement of the item) prior to entry into CFA and the weighted least squares with means and variance adjustment (WLSMV) estimator for categorical data was employed. This technique is consistent with previous CFAs establishing psychometric properties of the outcome scales (Achenbach and Rescorla 2000) and allows for increased power relative to models using indicators with empty cells.

Next, structural equation modeling (SEM) using the WLSMV estimator was employed to test the hypotheses that economic disadvantage would predict child internalizing and externalizing problems at age 3 years, and these relations would be mediated by maternal depressive symptoms, disrupted parenting, and home environments. In order to clarify the SEM findings, we conducted additional tests of indirect effects using the ‘indirect’ option in Mplus. Control variables were allowed to covary with each other and with economic disadvantage, and were entered as predictors of all other variables in the model (home environment, maternal depressive symptoms, parenting stress, harsh parenting, and child outcomes). In addition, our model took into account possible covariance among the two latent home environment constructs, the two latent parenting constructs, and the two latent child behavior constructs.

We tested whether child gender moderated the relationships shown in Fig. 1. In SEM analysis, the differences in chi-square values between a model that allows the parameters to vary among groups and a model that constrains the parameters to be equal across groups provides a test for moderation effects. When the difference is non-significant, there is no evidence of moderation. We did not evaluate ethnic differences in the processes linking economic disadvantage and children’s internalizing and externalizing problems because numbers of national origin groups in the Western and the different non-Western categories were too small for meaningful multiple-group analysis when considered separately. However, we included national origin as a control variable.

A separate analysis was run with father reports on internalizing and externalizing problems. Also in this analysis, missing data were estimated in order to use all available data in Mplus with FIML procedures. In addition, we conducted a separate analysis excluding those families who reported upward mobility (i.e., those families who were poor during pregnancy but were no longer poor when the child was 3 years of age).

Because chi-square values are sensitive to the sample size, we used the Comparative Fit Index (CFI), the Tucker-Lewis Index (TLI), and the Root Mean Square Error of Approximation (RMSEA) as our main indices of model fit (Browne and Cudeck 1993; Hu and Bentler 1999). For the CFI and TLI, values greater than 0.90 generally indicate reasonably good fit. For the RMSEA, values of 0.05 or lower indicate close fit, the range of 0.05 to 0.08 is interpreted as reasonable fit, the range of 0.08 to .10 as marginal fit, and values greater than 0.10 as unacceptable fit.

## Results

### Measurement Models

Before testing our structural model, we first performed confirmatory factor analyses to establish the validity of our proposed latent factors. The variable loadings on the latent factors and the fit indices are summarized in Table 1. For all five measurement models, a reasonably good fit to the data was found (see Table 1). In addition, all variable loadings on the hypothesized latent factors were strong and statistically significant. Thus, confirmatory factor analyses indicated that it was acceptable to employ the proposed latent constructs in the remaining analyses.

### Structural Model

**Fig. 2:**
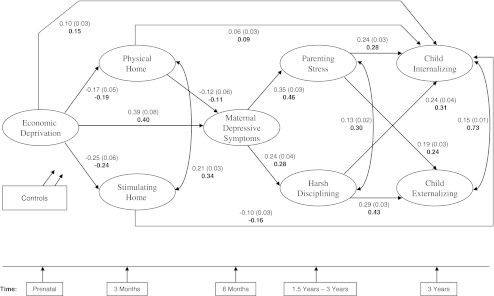
Unstandardized and standardized (*bold*) coefficient estimates (values given in parentheses are standard errors). All paths shown are statistically significant at the *p* < 0.05 level. All estimates include controls (age of child, gender, national origin, parity, maternal education, marital status and maternal age)

The results of our hypothesized model are presented as follows. Figure 2 presents the unstandardized and standardized path coefficients of the model. To enhance the readability of the figure, paths between control variables and outcomes are not shown in the model. For the same reason, only paths that were statistically significant at the *p* < 0.05 level are presented. Table 2 shows the total effect of economic disadvantage on internalizing and externalizing problem scores disaggregated into direct and indirect effects. All the estimates in Table 2 and Fig. 2 take into account the background variables of the families. The chi-square difference test for the multiple-group analysis of child gender was non-significant, *χ* ^2^(36) = 39.94, *p* = 0.2994, indicating no evidence of moderation.

**Table 2:** Direct, indirect, and total effects of economic disadvantage on children’s internalizing and externalizing problem scores

Economic disadvantage	Internalizing		Externalizing	
	*b(SE)* β		*b(SE)* β	
Total	0.175***	(0.033) **0.269**	0.068*	(0.028) **0.120**
Total direct	0.100**	(0.033) **0.154**	0.005	(0.029) **0.009**
Total Indirect	0.075***	(0.015) **0.115**	0.063***	(0.014) **0.111**
Via Physical home	−0.011	(0.006) −**0.016**	−0.005	(0.005) −**0.008**
Via Stimulating home	0.024*	(0.010) **0.037**	0.009	(0.008) **0.017**
Via Depression and harsh disciplining	0.022***	(0.006) **0.034**	0.027***	(0.007) **0.048**
Via Depression and parenting stress	0.033***	(0.008) **0.051**	0.025***	(0.007) **0.045**
Via Physical home, depression, and harsh disciplining	0.001	(0.001) **0.002**	0.001	(0.001) **0.003**
Via Physical home, depression, and parenting stress	0.002	(0.001) **0.003**	0.001	(0.001) **0.002**
Via Stimulating home, depression, and harsh disciplining	0.001	(0.001) **0.002**	0.002	(0.001) **0.003**
Via Stimulating home, depression, and parenting stress	0.002	(0.002) **0.003**	0.002	(0.001) **0.003**

*p < 0.05; **p < 0.01; ***p < 0.001

Structural equation modeling showed a good fit to the data for the model, CFI = 0.94, TLI = 0.96, RMSEA = 0.03, *χ* ^2^(350) = 1166.19. The model explained 36 % of the variance in children’s internalizing problem scores and 36 % of the variance in children’s externalizing problem scores. First, we examined the total effect of economic disadvantage on children’s internalizing and externalizing problem scores. Table 2 shows that children residing in economically deprived families are more likely to have internalizing, *β* = 0.27, *p* < 0.001, and externalizing, *β* = 0.12, *p* < 0.05, problems at age 3 years. Next, we examined the extent to which the home environment, maternal depressive symptoms and parenting played a mediating role in the associations between economic disadvantage and children’s internalizing and externalizing problems. The decomposition of the total effects of economic disadvantage is presented in Table 2 and shows that, for internalizing problems, both the direct, *β* = 0.15, *p* < 0.01, and the total indirect effects, *β* = 0.12, *p* < 0.001, are statistically significant. For externalizing problems, the total indirect, *β* = 0.11, *p* < 0.001, but not the direct effect, *β* = 0.01, *p* > 0.05, of economic disadvantage was significant. In the next paragraphs, we present the specific indirect effects of economic disadvantage on children’s internalizing and externalizing problems.

### Mediating Role of Home Environments

Figure 2 shows that economic disadvantage was negatively associated with the quality of the physical, *β* = -0.19, *p* < 0.01, and the stimulating home environment, *β* = -0.24, *p* < 0.001. For the physical as well as the stimulating home environment, a significant direct association with children’s internalizing problem scores was found. The association of the stimulating home environment was negative, and thus in the expected direction, *β* = -0.16, *p* < 0.01, whereas the association of the physical home environment was positive, *β* = 0.09, *p* < 0.05. Table 2 presents the specific direct effects of economic disadvantage on children’s outcomes. From this table, it is clear that the stimulating home environment, *β* = 0.04, *p* < 0.05, but not the physical home environment, is a mechanism through which economic disadvantage affects children’s internalizing problems.

### Mediating Role of Maternal Depressive Symptoms and Disrupted Parenting

Figure 2 further shows that economic disadvantage was positively associated with maternal depressive symptoms, *β* = 0.40, *p* < 0.001, which in turn was positively associated with parenting stress, *β* = 0.46, *p* < 0.001, and harsh disciplining, *β* = 0.28, *p* < 0.001. For both parenting stress and harsh disciplining, a significant association with internalizing and externalizing problem scores was found (see Fig. 2). The specific indirect effects presented in Table 2 confirm that maternal depressive symptoms, along with parenting stress or harsh disciplining are mechanisms through which economic disadvantage affects children’s internalizing and externalizing problem scores.

### Mediating Role of Home Environments, Maternal Depressive Symptoms, and Disrupted Parenting

In line with our hypothesis, the quality of the physical home environment was negatively associated with maternal depressive symptoms, *β* = -0.11, *p* < 0.05. However, estimates in Table 2 show that for both internalizing and externalizing problem scores, the specific indirect effects involving home environments, maternal depressive symptoms, and disrupted parenting simultaneously were non-significant. Thus, these pathways involving all constructs did not add to the prediction of children’s internalizing and externalizing problems above the effect each construct had.

### Father Report

Father and mother reports of child internalizing problems, *β* = 0.60, *p* < 0.001, and externalizing problems, *β* = 0.62, *p* < 0.001, were interrelated. Separate analysis with father reports on internalizing and externalizing problems yielded only small changes in effect sizes and patterns of statistical significance when compared with the model using mother reports (data not shown), with one notable exception. In the analysis with father reports, mother’s harsh disciplining was less strongly, albeit significantly, associated with children’s externalizing problems, *β* = 0.24, *p* < 0.001, and was unrelated to internalizing problems, *β* = 0.06, *p* > 0.05.

### Upward Mobility

A total of 98 children (4.5 %) in this sample were no longer poor at age 3 years, indicating upward mobility. Results were largely similar when excluding these 98 children from analysis, although the size of effect of the several associations was slightly larger (data not shown). However, the size of effect of the association between the stimulating home environment and internalizing problem scores was somewhat smaller in this analysis, *β* = -0.10, *p* = 0.110.

## Discussion

In this study we aimed to extend previous findings on how economic disadvantage affects young children’s emotional and behavioral problems to a non-American sample. Consistent with our hypotheses, economic disadvantage was associated with both internalizing and externalizing problems when children were as young as 3 years of age. Furthermore, as hypothesized, these associations were partially explained by maternal depressive symptoms, along with disrupted parenting including parenting stress and harsh disciplining. For internalizing, but not for externalizing problem scores, the quality of the stimulating home environment was an additional mechanism explaining the effect of economic disadvantage.

The pattern of the current results is largely consistent with those reported in a number of US studies (Linver et al. 2002; McLeod and Shanahan 1993; NICHD 2005; Pachter et al. 2006; Yeung et al. 2002) supporting the mediating roles of home environments, maternal emotional well-being, and parenting in the association between economic disadvantage and young children’s behavioral development. In this study, we found no direct effects of the physical home environment and the stimulating home environment on children’s externalizing problems, but rather a direct effect of a lower quality physical environment on the mother’s depressive symptoms. This extends the findings of Yeung et al. (2002) to a Dutch sample of young children. The current results also demonstrated support for the most basic propositions of the family investment model and those of the family stress model. That is, that economic disadvantage is negatively related to parental investments (e.g., the provision of learning toys in the home) that are expected to foster positive development for children, and that economic deprivation adversely affects the child’s development through its negative impact on maternal depression, which in turn diminishes or disrupts parenting skills.

Despite these consistent patterns, some considerable differences between the current study and related research can be noted. In their US sample, Yeung et al. (2002) observed that the physical environment of the home was indirectly related to children’s externalizing problems through its relation with maternal depressive symptoms and punitive parenting. Such an indirect effect was absent in the present study. This difference may be due to methodological differences in the assessment procedure of the Yeung et al. (2002) study and that of the current study. Unlike Yeung et al. (2002), who combined observed and interviewed reports, we assessed the home environment exclusively by observation, thereby limiting shared method variance bias. Furthermore, in the current study children’s home environments, maternal depressive symptoms, and developmental outcomes were all assessed at different points in time, whereas Yeung et al. (2002) assessed these constructs cross-sectionally. Related studies have often relied, at least in part, on cross-sectional designs. For example, Kiernan and Huerta (2008) assessed all their potential mediators, with the exception of maternal depression, at the same time as the child outcome measures. Given that the present study assessed the predictor, mediators, and outcomes at different points in time, tests of mediation are more rigorous. Indeed, of the five mediators being tested, only the assessment of maternal harsh disciplining was conducted at the same time as the outcomes.

Furthermore, previous research suggested that low income or socio-economic position is more closely related to externalizing than to internalizing problems (Amone-P’Olak et al. 2009; Kiernan and Huerta 2008; McLeod and Shanahan 1993; Yeung et al. 2002), while, if anything, the reverse was observed in the current study. Few studies, however, have investigated the associations between economic disadvantage and internalizing and externalizing problems in children as young as 3 years of age, as was done in the present study. In a study by Kiernan and Huerta (2008), economic disadvantage predicted both children’s internalizing and externalizing problems at age 3 years, showing relatively large effects on externalizing problems. However, any comparison must account for the different indicators used for both economic disadvantage and children’s internalizing and externalizing problems.

As with economic disadvantage, the current findings suggest that the stimulating home environment has a distinct effect on children’s internalizing problems, but less so on children’s externalizing problems. Therefore, our hypothesis that parental investments and family stress each independently explain the association between economic disadvantage and children’s behavioral development was confirmed in our analysis of internalizing but not in our analysis of externalizing problem scores. This finding may reflect the higher stability of internalizing than externalizing problems in young children (Achenbach and Rescorla 2000). The observed associations of economic disadvantage and home environments with children’s problem behavior were small to moderate in magnitude and thus their impact on externalizing problems may not have reached significance as externalizing problems are less stable at very young ages.

Much of the effect of economic disadvantage on externalizing problems was indirect rather than direct, indicating that this association is largely attributable to maternal emotional well-being and disrupted parenting. In contrast, the effect of economic disadvantage on children’s internalizing problems was direct rather than through the home environment, depressive symptoms, and disrupted parenting. This suggests that additional factors should be considered to explain this effect. This is in line with observations after a natural experiment that moved rural American families out of poverty (Costello, Compton, Keeler, & Angold, 2003). Moving out of poverty by sudden wealth significantly decreased children’s externalizing problems, but not internalizing problems (Costello et al., 2003). The authors suggested that internalizing problems may also be caused by some characteristics of poor families not directly related to poverty, such as a higher genetic loading for these conditions (Costello et al., 2003).

In order to achieve effective and efficient targeted intervention and prevention programs for young children and their families, additional research must delineate the processes by which economic disadvantage affects children’s emotional and behavioral problems. For example, nutrition and neighbourhood quality are likely to mediate these associations (Evans 2004; Martin et al. 2010). In this study, children’s home environments were observed in the presence of the primary caregiver, who is mostly the mother. Thus, our model focused on maternal emotional well-being. Other studies, however, found effects of paternal depressive symptoms on their parenting and their children’s developmental outcomes (Wilson and Durbin 2010). This gives rise to an important question for future research; namely, whether father’s emotional well-being and parenting contribute to the association between economic disadvantage and early-childhood problem behavior. Furthermore, the observed association between parental socio-economic status and children’s emotional and behavioral problems may be due to a third factor such as social selection (Conger and Donnellan 2007). More specifically, parents who are genetically predisposed to feelings of distress may have more difficulties in acquiring everyday financial necessities and have children who are also predisposed to distress and attendant behavioral problems.

Although the present study has a number of important strengths, its results must be interpreted within the context of several limitations. Firstly, the present study is population-based and maternal depression was assessed with a self-rating scale. Therefore, the results may not be easily generalizable to clinical populations. However, given that we were able to detect effects of maternal depressive symptoms in this study, it is likely that these effects would be more pronounced in populations at higher risk for psychopathology. Like other cohort studies, the Generation R Study is prone to selective drop-out. Our response analysis showed that selection occurred toward well functioning families with higher socio-economic status. Although it is certain that selective drop out has an impact on statistical power, a recent study and simulations on the Avon Longitudinal Study of Parents and Children (ALSPAC) sample showed that this does not need to affect the validity of regression models with regard to disruptive behavior (Wolke et al. 2009). In cases of selective drop out of families with lower socio-economic status some of the effects associated with economic disadvantage may be underestimated. It is possible that these associations are stronger in those who did not participate than in those who did. Furthermore, as is often the case within large-scale studies, data on child behavior relied on parental report. Consequently, associations between maternal depressive symptoms and child problem behavior may reflect a negative impact of maternal depression but could also be influenced by a tendency on the part of depressed mothers towards describing their children more negatively. However, we reduced possible reporter bias on the part of mothers in several ways. First, a temporal sequence was established and maternal depressive symptoms were assessed several years prior to the assessment of the outcome. Also, information on the home environment was not obtained by self-report of mothers but relied exclusively on observations by trained research nurses. Lastly, both mothers and fathers reported on child behavioral problems, and the associations were found to be largely consistent across informants. A notable exception was that in the analysis with father report, mother’s harsh disciplining was less strongly associated with children’s externalizing problems and was unrelated to internalizing problems. Of all constructs, this harsh disciplining construct was the only one assessed cross-sectionally with internalizing and externalizing problems. This study thus underscores the importance of temporal sequences in research on family processes and children’s behavioral development. However, the fact that a temporal sequence was established does not mean that the relationships between these variables are necessarily unidirectional. For instance, a less-optimal home environment may lead to maternal depressive symptoms but the reverse could also be true. Finally, the family income and home environment variables were measured very early in life and are likely to fluctuate over time. Particularly for young parents, there may be upward mobility. We addressed this by conducting a separate analysis excluding those children and their families who had moved out of poverty at the age of 3 years. Results were found to be largely consistent. Data on home environments, however, were obtained only once and early in life. However, it has been documented that by the age of 6 months, many children are already able to provoke encouragement and attention from their parents, suggesting mutual influence of child and environment (Bradley 1993, 1994; Zeenah et al. 1997).

Young children constitute an important group to policy makers and intervention designers. As early as the first few years of life, associations between economic disadvantage and children’s developmental problems are observable and the future burden of mental health problems may be preventable by the use of well-designed interventions based on empirical research. By investigating possible mechanisms underlying the harmful impact of economic disadvantage on children’s behavioral and emotional development, the findings of the current study have implications for early intervention programs. In a publicly funded health-care system such as the Netherlands, children residing in economically disadvantaged households are at increased risk of developing emotional and behavioral problems. The present study supports earlier US research indicating that interventions that focus solely on raising income levels may not adequately address problems in the family processes that emerge as a result of economic disadvantage (Linver et al. 2002; McLeod and Shanahan 1993; NICHD 2005; Pachter et al. 2006; Yeung et al. 2002). Policies to improve the mental health of mothers with young children but also their home environments are needed to change the economic gradient in child behavior.

This study contributes to the literature by unraveling pathways between economic disadvantage, children’s home environments, maternal depressive symptoms, disrupted parenting, and child emotional and behavioral outcomes at the age of 3 years. We conclude that for both children’s internalizing and externalizing problems, mechanisms explaining the effect of economic disadvantage include those of maternal depressive symptoms, along with disrupted parenting. For children’s internalizing but not externalizing problems, the stimulating learning environment of the home explained part of the effect of economic disadvantage.
